# Humic Acid-Derived Porous Carbon as Peroxymonosulfate Activator for Phenol Removal

**DOI:** 10.3390/molecules31060975

**Published:** 2026-03-13

**Authors:** Mingqi Sun, Xinning Sun, Jiuling Huang, Hao Dong, Zhongming Guo, Jianjun Qu, Jianhua Xiao, Xiaoli Zhu, Baoshou Shen

**Affiliations:** 1 Shaanxi Key Laboratory of Earth Surface System and Environmental Carrying Capacity, College of Urban and Environmental Sciences, Northwest University, Xi’an 710127, China; 15269201728@163.com (M.S.); sunxinning71@163.com (X.S.); 202232915@stumail.nwu.edu.cn (J.H.); donghao_0105@163.com (H.D.);; 2 Xi’an Key Laboratory of Environmental Simulation and Ecological Health in the Yellow River Basin, Xi’an 710127, China; 3 Shaanxi Key Laboratory for Carbon Neutral Technology, Carbon Neutrality College (Yu Lin), Northwest University, Xi’an 710127, China; 4 Key Laboratory of Ecological Safety and Sustainable Development in Arid Lands, Northwest Institute of Eco-Environment and Resources, Chinese Academy of Sciences, Lanzhou 730000, China; xiaojh@lzb.ac.cn

**Keywords:** phenol, humic acid, nitrogen doping, peroxymonosulfate activation

## Abstract

To enable the efficient and environmentally benign treatment of phenol-containing wastewater, a nitrogen-doped porous carbon material (denoted as 900-CN) was synthesized via high-temperature annealing of a composite composed of humic acid (HA) and g-C_3_N_4_. The as-prepared materials were characterized, and their catalytic performance in activating peroxymonosulfate (PMS) for phenol degradation was investigated. The results demonstrate that g-C_3_N_4_ acts as a layered template; upon high-temperature annealing, it gradually evolves into a highly wrinkled and porous architecture. This morphology substantially increases the specific surface area, thereby facilitating pollutant removal. PMS formed metastable surface complexes on 900-CN, enabling concomitant electron transfer. Concurrently, functional groups on the HA-derived carbon reacted with PMS to generate singlet oxygen (^1^O_2_), a highly oxidative species that markedly enhanced phenol degradation. The 900-CN composite achieved complete phenol removal (100%) within 60 min. Variations in reaction temperature (20–50 °C) and initial pH (2–10) exhibited negligible influence on the performance of the 900-CN/PMS system. Reactive species in the 900-CN/PMS/phenol system included •OH, SO_4_^•−^, O_2_^•−^, and ^1^O_2_, indicating that phenol degradation occurred through combined radical and non-radical pathways. These findings highlight the strong potential of 900-CN as a promising catalyst for the treatment of phenolic wastewater.

## 1. Introduction

In recent years, water contamination has become a major global challenge, posing serious threats to ecosystem integrity and the long-term sustainability of human development. The widespread occurrence of hazardous organic compounds, particularly phenolic substances, in water sources and industrial effluents constitutes a pressing global environmental problem [1,2,3]. Phenolic compounds are widely recognized as representative toxic pollutants in aquatic environments due to their high solubility and carcinogenic properties. These substances are frequently detected in industrial effluents discharged from various sectors, including petroleum refining, petrochemical processing, pharmaceutical manufacturing, resin and polymer production, plastics fabrication, papermaking, and wood-related industries. As one of the most prevalent and widely distributed phenolic pollutants, phenol warrants particular attention [4,5]. Therefore, the development of more efficient and environmentally sustainable technologies for treating phenol-laden wastewater is urgently required.

Advanced oxidation processes (AOPs) offer distinct advantages for the treatment of highly contaminated industrial wastewater containing toxic, hazardous, refractory, and non-biodegradable organic pollutants, and thus occupy a pivotal position in wastewater treatment technologies [6,7]. These processes are widely employed for the mineralization of persistent organic contaminants via radical-mediated oxidation pathways [8,9,10]. Sulfate radical-based advanced oxidation processes (SR-AOPs) have demonstrated remarkable efficacy in degrading recalcitrant pollutants [11,12], such as phenolic compounds, pharmaceuticals, dyes, endocrine-disrupting chemicals (EDCs), chlorinated organics, and antibiotics [13,14,15,16,17,18]. In addition to their application in pollutant degradation, persulfates have also been employed in selective oxidation reactions when activated by carbonaceous materials, where non-radical pathways such as ^1^O_2_-mediated oxidation play an important role. Recent studies have demonstrated that carbon-based catalysts can facilitate PMS activation through surface-bound intermediates, enabling controlled and selective oxidation processes [19,20,21]. Peroxymonosulfate (PMS) is a cost-effective precursor for the generation of sulfate radicals (SO_4_^•−^) [22,23]. Compared to hydroxyl radicals (•OH, 1.95–2.80 V) [24], SO_4_^•−^ exhibits a higher redox potential, greater reaction selectivity [25], and a longer half-life. Furthermore, SO_4_^•−^ remains effective over a broad pH range [26].

PMS can be activated via various pathways, including heat treatment, ultrasonic irradiation, photochemical/radiation activation, alkaline conditions, transition-metal catalysis, and carbon-based activators [13]. Among these approaches, activation by carbon-based materials represents an environmentally benign, stable, and energy-efficient strategy. Since its identification in 2009 as a metal-free polymeric semiconductor capable of driving photocatalytic hydrogen production, graphitic carbon nitride (g-C_3_N_4_), a representative synthetic polymer, has attracted increasing interest in the fields of chemical and materials research [27,28]. This is attributed to its cost-effectiveness, ease of synthesis, robust stability, and unique physicochemical properties [29]. In their comprehensive review, Zhang et al. summarized the synthesis routes, approaches for structural tuning, and visible-light-driven photocatalytic applications of g-C_3_N_4_ in the degradation of organic contaminants [30]. Wen et al. proposed innovative design strategies for g-C_3_N_4_-based nanomaterials and systematically discussed their applications in photocatalytic water splitting and organic pollutant degradation [31]. Furthermore, Liu et al. systematically investigated the effects of heteroatom doping in g-C_3_N_4_ on its optical absorption properties, charge carrier lifetime, and charge separation/transport efficiency [32]. Considerable efforts have been devoted to the development and tailoring of g-C_3_N_4_ and its nanostructures, as well as exploring their applications in metal-ion adsorption and the photocatalytic removal of organic contaminants. However, pristine g-C_3_N_4_ suffers from inherent limitations including low specific surface area, severe charge carrier recombination, inefficient photogenerated charge separation, and unsatisfactory quantum efficiency [33,34]. To overcome these drawbacks, various approaches has been proposed to improve the catalytic performance. Utilizing g-C_3_N_4_ as a nitrogen precursor offers dual advantages: (i) it eliminates the need for post-synthetic template removal, and (ii) it generates in situ gaseous products during thermal decomposition, which facilitates the formation of porous architectures in carbon matrices and thereby significantly increases the specific surface area [35,36,37]. Humic acid (HA), an abundant natural material containing diverse functional groups (e.g., carboxyl, phenolic, and hydroxyl), serves as an excellent precursor for carbon-based materials [38,39]. These HA-derived carbons exhibit remarkable adsorption capabilities for both metal ions and organic pollutants owing to their inherent surface chemistry [40,41]. Jiang et al. demonstrated that HA significantly enhances the permanganate oxidation of triclosan (TCS) in the pH range of 5–7, with the reaction efficiency increasing by 40–65% compared to HA-free systems [42]. Currently, HA-derived carbonaceous materials have found diverse applications across multiple domains, exhibiting satisfactory performance in various functional contexts [43,44]. Although studies on HA-based catalytic materials remain relatively scarce, their distinctive physicochemical characteristics provide a sound basis for the development of advanced carbon-derived catalysts.

In this study, nitrogen-doped porous carbon composites (denoted as 900-CN) were synthesized via high-temperature annealing of g-C_3_N_4_ and HA for phenol removal. The 900-CN material was systematically characterized to elucidate the evolution of its microstructure, pore architecture, and surface functional groups during the carbonization process. The influence of major operating variables on the removal performance was systematically evaluated, including catalyst loading (0.2–1.0 g/L), PMS dosage (0.5–3.0 mM), solution pH (2–10), reaction temperature (20–50 °C), and initial phenol concentration (50–300 mg/L). The reaction mechanism was elucidated through radical quenching experiments and electron paramagnetic resonance (EPR) spectroscopy, which identified dominant reactive oxygen species (•OH, SO_4_^•−^, O_2_^•−^, and ^1^O_2_). Intermediate products were analyzed by GC-MS to propose degradation pathways. Compared with previously reported biomass-derived carbon catalysts, the present work introduces a dual-template carbonization strategy by integrating g-C_3_N_4_ and humic acid, enabling simultaneous pore structure optimization and in situ nitrogen incorporation, which enhances the non-radical PMS activation pathway.

## 2. Materials and Methods

### 2.1. Chemicals

All reagents were of analytical or chromatographic grade and were used as received without additional purification: Phenol (C_6_H_5_OH, 99.5%), humic acid (HA), urea (CO(NH_2_)_2_), acetonitrile (C_2_H_3_N, HPLC grade), methanol (CH_3_OH, HPLC grade), ethanol (95%), ammonium hydroxide (NH_3_·H_2_O), potassium monopersulfate triple salt (PMS, 2KHSO_5_·KHSO_4_·K_2_SO_4_), sodium hydroxide (NaOH), hydrochloric acid (HCl), tert-butanol (C_4_H_10_O), furfuryl alcohol (C_5_H_6_O_2_), p-benzoquinone (C_6_H_4_O_2_), 5,5-dimethyl-1-pyrroline N-oxide (DMPO), and 2,2,6,6-tetramethylpiperidine (TEMP). These reagents were purchased from Xi’an Ruilijie Experimental Equipment Co., Ltd (Xi’an, China).

### 2.2. Preparation of Materials

A specified amount of urea was weighed and transferred into a covered alumina crucible, which was then placed in a muffle furnace. The furnace was heated to 550 °C at a rate of 5 °C/min and maintained at this temperature for 4 h. After natural cooling to room temperature, the resulting g-C_3_N_4_ was collected and stored in a sealed container for subsequent use. HA and g-C_3_N_4_ were mixed at mass ratios of 1:3, 1:2, 1:1, and 2:1. Each mixture was transferred to a beaker, followed by the addition of 50 mL of ultrapure water to form a uniform dispersion. Subsequently, 10 mL of ammonia solution was added, and each mixture was stirred at 600 rpm for 12 h. The resulting suspensions were dried in an oven at 80 °C for 12 h, ground into powders, and transferred to a tube furnace. The samples were first annealed at 300 °C for 1 h under a nitrogen atmosphere with a heating rate of 5 °C/min. Subsequently, they were further heated to 700, 800, or 900 °C, respectively, and maintained at each target temperature for 1 h. After natural cooling to room temperature naturally, the black products were collected and repeatedly washed with hydrochloric acid and ultrapure water until the pH became neutral. Finally, the samples were dried in a vacuum oven at 80 °C overnight, yielding nitrogen-doped porous carbon materials with varying mass ratios and annealing temperatures.

### 2.3. Characterization of Materials

The surface morphology of the prepared materials was observed using scanning electron microscopy (SEM, SU-8010, Hitachi, Saitama, Japan). Fourier transform infrared spectroscopy (FTIR, TENSOR 27, Bruker, Ettlingen, Germany) was employed to identify surface functional groups and their variations. Specific surface area, pore size distribution, and nitrogen adsorption–desorption isotherms were measured using a Brunauer–Emmett–Teller surface area analyzer (BET, ASAP 2460, Micromeritics, Norcross, GA, USA). The crystalline structure of the samples was investigated by X-ray diffraction (XRD, D8 Advance, Bruker, Germany) with Cu Kα radiation. X-ray photoelectron spectroscopy (XPS, K-Alpha, Thermo Scientific, Waltham, MA, USA) was used to analyze the elemental composition and chemical valence states. Electron paramagnetic resonance spectroscopy (EPR, ELEXSYS-II E500, Bruker, Germany) was employed to detect reactive radical species generated in the activated persulfate system. Degradation intermediates were identified by gas chromatography–mass spectrometry (GC–MS, 7890B-7000D, Agilent, Santa Clara, CA, USA), and possible degradation pathways were proposed accordingly.

### 2.4. Experimental Procedures

A phenol solution with an initial concentration of 20 mg/L was prepared as a wastewater model. In a conical flask, 25 mg of catalyst was added, followed by 30 mg of PMS (KHSO_5_·0.5KHSO_4_·0.5K_2_SO_4_). Subsequently, 100 mL of the phenol solution was introduced without adjusting the initial pH. The flask was shaken at 25 °C on an orbital shaker at 180 r/min. Aliquots were withdrawn every 15 min. Each sample (2 mL) was filtered through a 0.22 μm polyethersulfone syringe filter. Next, 1 mL of the filtrate was transferred into a liquid chromatography vial and promptly combined with 0.5 mL of methanol to halt the reaction. The phenol concentration and removal efficiency were quantified by high-performance liquid chromatography based on the peak area. All experiments were performed in triplicate, and a blank control was included.

### 2.5. Analytical Methods

Phenol concentrations were quantified using high-performance liquid chromatography (HPLC) equipped with a Shim-pack VP-ODS C18 column (150 mm × 4.6 mm, Shimadzu, Kyoto, Japan). The analysis was performed under isocratic conditions with UV detection at 270 nm and a column temperature of 30 °C. The mobile phase consisted of ultrapure water (A) and acetonitrile (B) at a volume ratio of 7:3. The injection volume was set at 10 μL, with a flow rate maintained at 1.0 mL/min. Under these conditions, phenol exhibited a retention time of approximately 5.6 min.

## 3. Results and Discussion

### 3.1. The Characterization Results of the Material’s Morphological Structure

**Figure 1:**
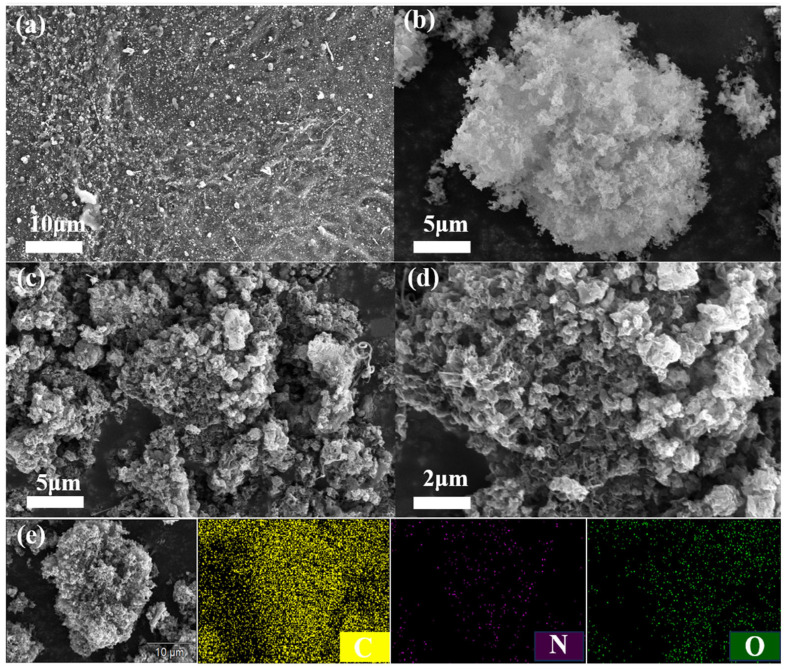
(**a**) SEM image of humic acid-derived carbon (900-HA); (**b**) SEM image of g-C_3_N_4_; (**c**,**d**) SEM images of 900-CN; (**e**) Elemental mapping image of 900-CN.

Figure 1 presents the SEM images of humic acid-derived carbon 900-HA, g-C_3_N_4_ and 900-CN-derived porous carbon. As shown in Figure 1a, the surface of 900-HA exhibits a rough, loose, and irregular granular morphology with no discernible pore structure. Figure 1b reveals that g-C_3_N_4_ possesses a thin, wrinkled lamellar structure with tightly stacked and aggregated layers. This three-dimensional porous architecture, formed via the curling and layer-by-layer assembly of two-dimensional graphitic-like C_3_N_4_ nanosheets, is well suited for use as a template in material synthesis. As shown in Figure 1c,d, the 900-CN material—a nitrogen-doped porous carbon synthesized at 900 °C—displays a rough and porous surface with a well-defined pore architecture. This morphological transformation confirms that high-temperature annealing of the HA/g-C_3_N_4_ mixture substantially alters the carbon material’s microstructure. In this process, g-C_3_N_4_ acts as a sacrificial template; high-temperature annealing induces the formation of a highly wrinkled and porous structure. This morphology enhances the specific surface area, thereby facilitating pollutant removal. Moreover, g-C_3_N_4_ serves as a nitrogen-rich precursor, enabling the resulting carbon material to exhibit both a porous morphology and in situ nitrogen doping, as illustrated in Figure 1e.

**Figure 2:**
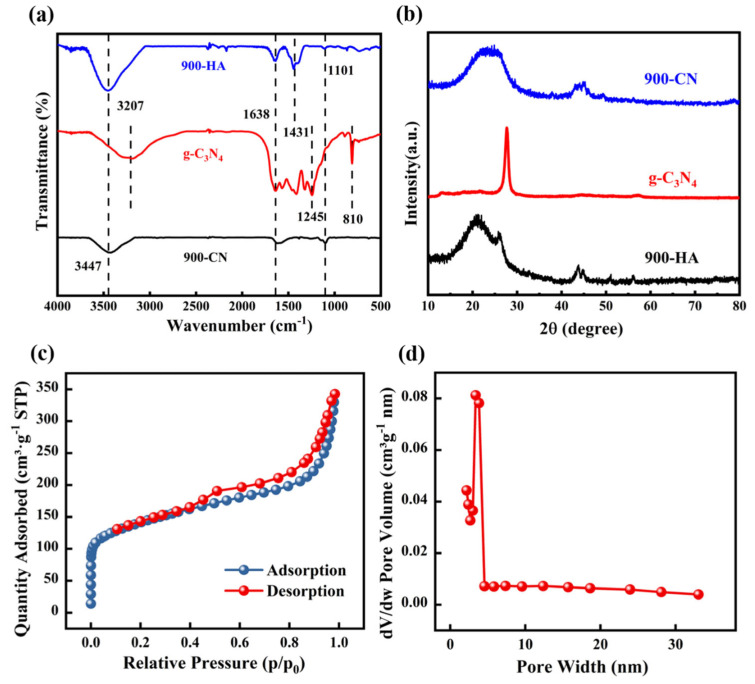
(**a**) FTIR spectra of 900-HA, g-C_3_N_4_, and 900-CN; (**b**) XRD patterns of 900-HA, g-C_3_N_4_, and 900-CN; (**c**) N_2_ adsorption-desorption isotherm of 900-CN; (**d**) pore size distribution profile of 900-CN.

FTIR was employed to investigate the functional group changes before and after material compositing. As shown in Figure 2a, both 900-HA and 900-CN exhibit a broad absorption band centered at 3447 cm^−1^, which is attributed to the O–H stretching vibration and may also be associated with the CO–NH stretching vibration in amino groups. The characteristic peak near 1638 cm^−1^ is attributed to the C=O stretching vibration, whereas the peak at approximately 1101 cm^−1^ is associated with the C–O stretching vibration. For 900-HA, the distinct peak at 1431 cm^−1^ is ascribed to the C–H bending vibration. For g-C_3_N_4_, the absorption band in the 3000–3500 cm^−1^ region arises from the stretching vibrations of O–H and N–H [45]; the peaks between 1245 and 1638 cm^−1^ correspond to the C–N stretching vibrations within the 3-s-triazine rings (C_6_N_7_) [46]; and the peak near 810 cm^−1^ is attributed to the breathing vibration mode of the heptazine ring [47]. These features collectively confirm the successful synthesis of g-C_3_N_4_. The FTIR spectrum of 900-CN-derived porous carbon shows no characteristic peak near 810 cm^−1^ corresponding to heptazine rings, indicating that nitrogen was fully incorporated into the carbon matrix with no residual g-C_3_N_4_. This result further validates the use of g-C_3_N_4_ as a sacrificial template. Figure 2b presents the XRD patterns of 900-HA, g-C_3_N_4_, and 900-CN. The diffraction patterns of 900-HA and 900-CN are highly consistent, both exhibiting two broad reflections at 22.53° and 43.01°, which are assigned to the (002) and (100) planes of carbon, respectively. These reflections are characteristic of the interlayer stacking of graphite-like structures [48]. In contrast, pristine g-C_3_N_4_ exhibits a sharp peak at 2θ = 27.13°, indexed to the (002) plane, which arises from the stacking of aromatic units within its layered framework [49].

Figure 2c presents the N_2_ adsorption–desorption isotherm of 900-CN. A distinct hysteresis loop is observed in the relative pressure (P/P_0_) range of 0.4–1.0, corresponding to a typical Type IV isotherm and indicating the presence of a mesoporous structure. According to the IUPAC classification, this hysteresis loop is categorized as Type H3. The absence of a distinct adsorption plateau in the Type H3 isotherm suggests the presence of irregular, slit-shaped pore structures in the 900-CN material. Figure 2d displays the pore size distribution of 900-CN, revealing a predominant distribution of mesoporous characteristics. The detailed textural parameters, including specific surface area, total pore volume, and pore size distribution, are summarized in Table 1. The nitrogen-doped porous carbon material 900-CN was synthesized by annealing a 1:2 mixture of HA and g-C_3_N_4_ at 900 °C. Remarkably, 900-CN exhibits a significantly enhanced specific surface area of 507.06 m^2^/g, far exceeding those of 900-HA (15.10 m^2^/g) and g-C_3_N_4_ (45.13 m^2^/g) obtained under identical annealing conditions. Concurrently, the total pore volume increases to 0.53 cm^3^/g while the average pore size decreases to 4.18 nm. This textural evolution is attributed to the complete decomposition of g-C_3_N_4_ during annealing, which leads to contraction of the carbon framework. The dual function of g-C_3_N_4_—serving as both a sacrificial template and a nitrogen-rich precursor—confers three distinct advantages to the resulting 900-CN material: well-developed porosity, a substantially increased specific surface area, and in situ nitrogen doping within the carbon matrix. The drastic increase in surface area of 900-CN can be attributed to the sacrificial-template role of g-C_3_N_4_ during high-temperature annealing. The thermal decomposition of g-C_3_N_4_ generates gaseous species that promote pore formation and induce carbon framework rearrangement, resulting in enhanced porosity and surface area. Although direct reports on the co-carbonization of humic acid and g-C_3_N_4_ for pollutant removal remain limited, similar pore-forming effects of g-C_3_N_4_-derived carbon materials have been widely reported in the literature [50,51,52,53].

**Figure 3:**
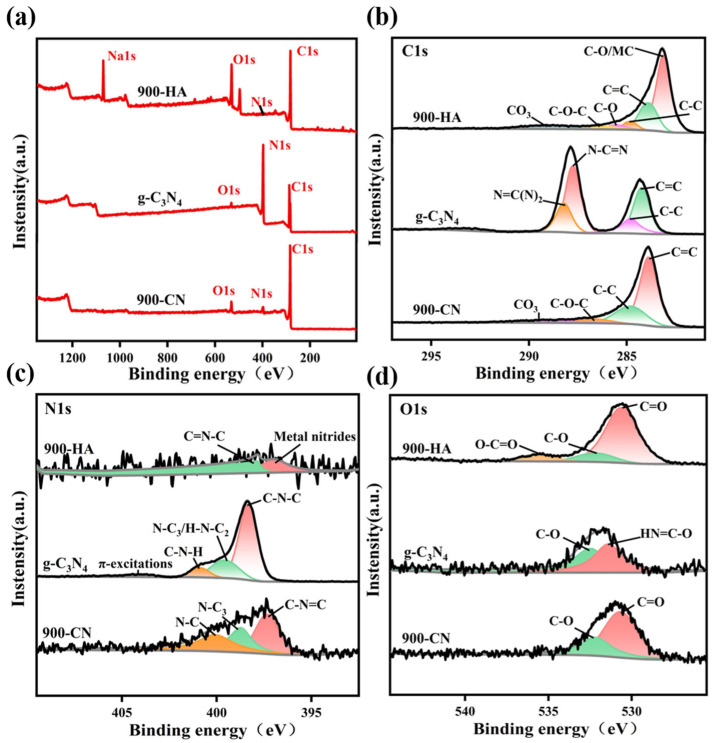
Presents the XPS analysis of 900-HA, g-C_3_N_4_, and 900-CN: (**a**) survey spectra, (**b**) C 1s, (**c**) N 1s, and (**d**) O 1s.

XPS was employed to characterize the surface chemical compositions of 900-HA, g-C_3_N_4_, and 900-CN (Figure 3). The survey spectra (Figure 3a) reveal that C, N, and O are the primary constituent elements in all three materials. After compositing, the 900-CN composite exhibits a marked increase in carbon content relative to its individual precursors. During high-temperature annealing, nitrogen from g-C_3_N_4_ was incorporated into the 900-HA matrix, resulting in an increased content in 900-CN. Concurrently, partial oxygen loss was observed during the thermal treatment process. Figure 3b presents the high-resolution C 1s spectra. Among the three materials, 900-HA exhibits the highest carbon content and the most complex spectral profile. The dominant peak at 283.2 eV is assigned to C-O bonds and/or metal carbides (MCs). The features at 283.88 eV (900-HA), 284.24 eV (g-C_3_N_4_), and 283.88 eV (900-CN) are attributed to sp^2^-hybridized C=C bonds, confirming the presence of graphitic structures in all three samples [54]. All three materials exhibit a characteristic peak at 284.80 eV, which arises from carbon atoms in C-C bonds. After composite formation, the C 1s binding energy of 900-CN exhibits no significant shift relative to its precursors, whereas the enhanced intensity of the C=C peak (sp^2^ hybridization) suggests improved graphitization during thermal annealing. Notably, the peak at 287.75 eV in g-C_3_N_4_ is assigned to carbon atoms within N-C=N functional groups, which is consistent with its triazine-based structure [55]. The C at 288.21 eV can be attributed to the sp^2^ hybridized carbon atoms bonded with N in the aromatic ring (N=C(N)_2_) [56]. Figure 3c presents the N 1s spectrum. The nitrogen content in 900-HA was negligible. For g-C_3_N_4_, the N 1s peaks at 398.34 eV and 400.88 eV correspond to sp^2^-hybridized nitrogen in C–N–C units [57] and amino-type nitrogen in C–N–H groups [58], respectively. In addition, the peak at approximately 404.18 eV is assigned to π-π* transitions associated with charging or π-excitation effects [55]. The peak at approximately 399.51 eV has been assigned to tertiary nitrogen species, such as N-C_3_ or H-N-C_2_ [59]. For 900-CN, the peaks at 397.33 eV and 398.72 eV are attributed to sp^2^ nitrogen (C=N-C) within the triazine framework and quaternary nitrogen coordinated with three carbon atoms in the aromatic structure, respectively [60,61]. The peak at 400.07 eV is attributed to sp^3^ N-C bonding [62].

Figure 3d presents the O 1s spectrum. The peak corresponding to HN=C–O in g-C_3_N_4_ is attributed to oxygen-containing intermediates formed during the incomplete pyrolysis of urea [63]. In 900-HA, a peak corresponding to the O–C=O group was observed at 535.52 eV, which was absent in the spectrum of 900-CN. This absence is likely attributed to the transformation of O–C=O groups into C=O and C–O species at elevated temperatures [62].

### 3.2. Degradation Performance and Influencing Factors of the 900-CN/PMS/Phenol System

**Figure 4:**
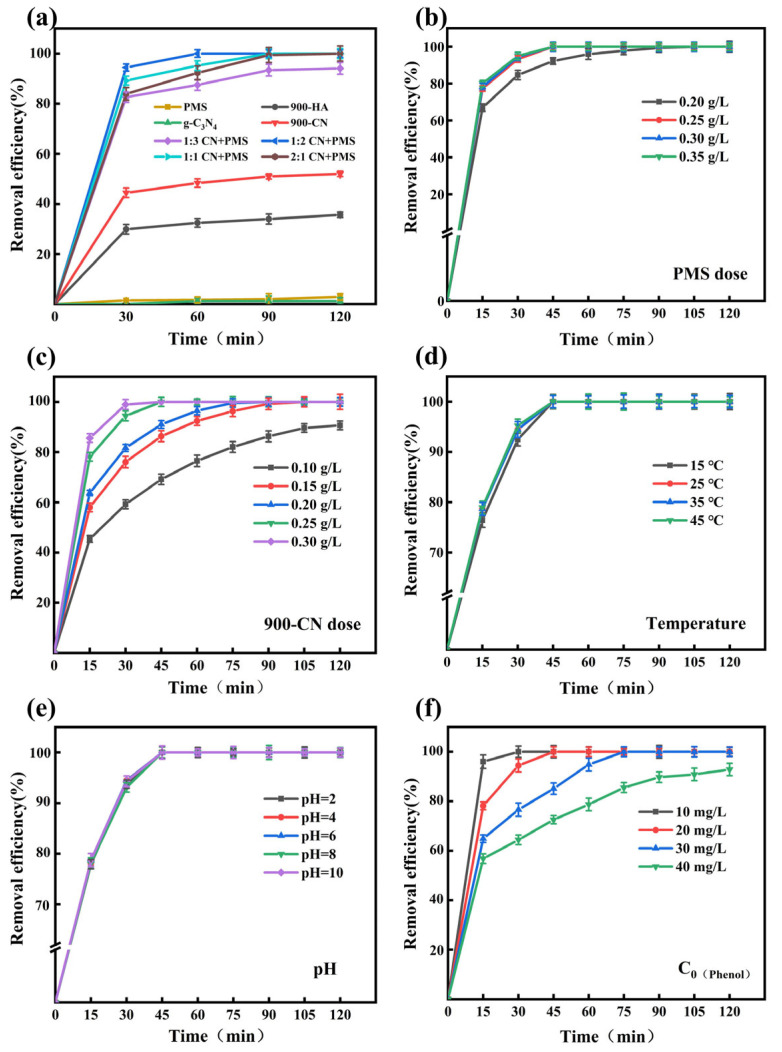
(**a**) The removal effects of phenol by different systems; (**b**) dosage of PMS; (**c**) dosage of materials; (**d**) different reaction temperatures; (**e**) different pH; (**f**) the influence of pollutant concentration on the removal effect of phenol.

Figure 4a illustrates that PMS or g-C_3_N_4_ alone exhibited negligible phenol removal in the absence of a catalyst. In the absence of PMS, 52.03% of phenol was removed via adsorption by 900-CN alone. When 900-CN and PMS were used together, the phenol removal efficiency reached 94.10–100% within 120 min, demonstrating a marked enhancement. Specifically, when 900-CN was prepared with a HA: g-C_3_N_4_ mass ratio of 1:2, phenol was completely degraded within 60 min, exhibiting the highest efficiency.

As illustrated in Figure 4b, the phenol degradation rate increased gradually with increasing PMS dosage, and complete degradation was achieved within 120 min. With increasing PMS dosage, the catalyst reacted with ample oxidant, which provided abundant active sites and enhanced the reaction rate. However, an excessive PMS concentration generated an overabundance of radicals, which could self-quench or react with excess persulfate, leading to a decreased degradation rate [64]. As illustrated in Figure 4c, the removal rate increased gradually with increasing catalyst dosage. When the catalyst dosage was 0.25 g/L, phenol was completely removed within 45 min, and the degradation rate improved significantly. As illustrated in Figure 4d, with increasing reaction temperature from 15 °C to 45 °C, the reaction rate increased to some extent; however, complete phenol removal was still achieved within 45 min. This is likely due to the relatively low activation energy required for 900-CN to activate PMS for phenol removal, enabling effective removal even at lower temperatures. As illustrated in Figure 4e, when the initial pH was 2, 4, 6, 8, or 10, phenol was completely removed within 45 min, with no significant difference in degradation time or rate. This indicates that phenol degradation in this system was essentially unaffected by the initial solution PH. Compared to traditional materials, which are often constrained by pH limitations, 900-CN exhibits superior performance and stability. As illustrated in Figure 4f, the phenol degradation rate decreased with increasing initial phenol concentration. When the initial phenol concentration was 10 mg/L, 20 mg/L, and 30 mg/L, phenol was completely removed; however, the degradation rate was faster at lower concentrations. When the initial phenol concentration was 40 mg/L, the removal efficiency of phenol by 900-CN-activated PMS was 92.84%. This may be attributed to the occupation of active sites by competing species at higher pollutant concentrations, which resulted in a decreased removal rate. Alternatively, as the reaction progressed, substantial amounts of phenolic degradation intermediates were generated, which competed with phenol for reactive radicals.

### 3.3. Quenching Experiments and EPR Detection

**Figure 5:**
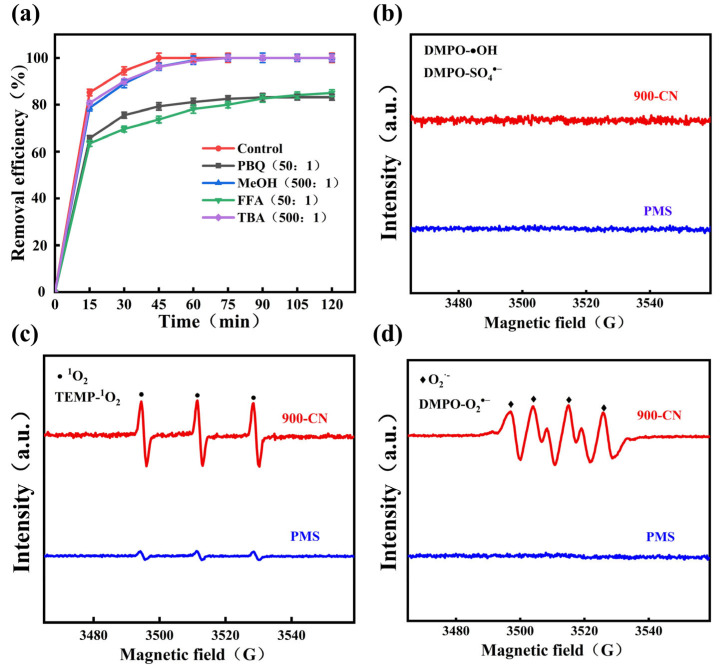
Effects of different quenchers on phenol removal by 900-CN-activated PMS, (**a**) 900-CN-activated PMS system; (**b**) DMPO-•OH and DMPO-SO_4_^•−^; (**c**) TEMP-^1^O_2_; (**d**) DMPO-O_2_^•−^. (Phenol = 20 mg/L, catalyst dosage 0.25 g/L, PMS dosage 0.25 g/L, without adjusting the initial pH value and reaction temperature).

The main radicals involved in the PMS-activated 900-CN system for phenol removal were identified through radical scavenging experiments. As shown in Figure 5a, when a large amount of MeOH and TBA were added, the inhibition effect on phenol degradation was still not significant, indicating that there were only trace amounts of SO_4_^•−^ and •OH radicals in the system. When PBQ and FFA were added, the phenol removal efficiency decreased to 83.21% and 85.17%, respectively. This suggests that O_2_^•−^ and ^1^O_2_ were present in the system and their effects were stronger than those of SO_4_^•−^ and •OH radicals. To further confirm the generation of reactive species, electron paramagnetic resonance (EPR) spectroscopy was employed. As shown in Figure 5b–d, no characteristic signals corresponding to •OH, SO_4_^•−^ or O_2_^•−^ were detected when PMS was added alone. However, a weak signal attributable to ^1^O_2_ was observed, arising from the minor self-decomposition of PMS. When both PMS and 900-CN were introduced, no signals corresponding to SO_4_^•−^ or •OH were detected, whereas the signals attributable to ^1^O_2_ and O_2_^•−^ were substantially intensified. Consistent with the radical quenching experiments, these EPR results indicate that •OH and SO_4_^•−^ were present in negligible amounts, whereas ^1^O_2_ and O_2_^•−^ were generated in substantial quantities.

Collectively, these findings demonstrate that both radical-driven and non-radical pathways are critically involved in the degradation of phenol. The possible reaction pathways are as follows: (1) PMS is adsorbed onto the active sites of 900-CN, and the abundant functional groups on the material surface can activate PMS to generate •OH and SO_4_^•−^, as shown in Equations (1)–(3). Simultaneously, HSO_5_^−^ hydrolyzes to generate H_2_O_2_, as shown in Equation (4), which further decomposes to yield O_2_^•−^, as shown in Equations (5) and (6). The combined action of •OH, SO_4_^•−^ and O_2_^•−^ constitutes the radical pathway for phenol removal. (2) PMS is adsorbed onto the surface of 900-CN, forming an unstable complex with concomitant electron transfer, which promotes the degradation of phenol. Concurrently, C-O functional groups on the surface of HA-derived carbon are oxidized to C=O, which catalyzes the decomposition of PMS to generate singlet oxygen (^1^O_2_) [65,66,67], as shown in Equations (7) and (8). The generated ^1^O_2_ exhibits strong oxidative activity. This represents the non-radical pathway.

(1) $$900-\mathrm{CN}+\mathrm{HS}{O_{5}}^{-}\to S{O_{4}}^{2-}+•\mathrm{OH}$$

(2) $$900-\mathrm{CN}+\mathrm{HS}{O_{5}}^{-}\to S{O_{4}}^{•-}+OH^{-}$$

(3) $$S{O_{4}}^{•-}+H_{2}O\to S{O_{4}}^{2-}+•\mathrm{OH}+H^{+}$$

(4) $$\mathrm{HS}{O_{5}}^{-}+H_{2}O\to \mathrm{HS}{O_{4}}^{-}+H_{2}O_{2}$$

(5) $$H_{2}O_{2}+•\mathrm{OH}\to H{O_{2}}^{•}+H_{2}O$$

(6) $$H{O_{2}}^{•}\to {{O_{2}}^{•}}^{-}+H^{+}$$

(7) $$\mathrm{Phenol}+\mathrm{HS}{O_{5}}^{-}+e^{-}\to intermediate+S{O_{4}}^{2-}$$

(8) 900−CNC=O+HSO5−+OH→O21+H2O+SO42−

### 3.4. Degradation Pathways and Mechanisms in the 900-CN/PMS/Phenol System

**Figure 6:**
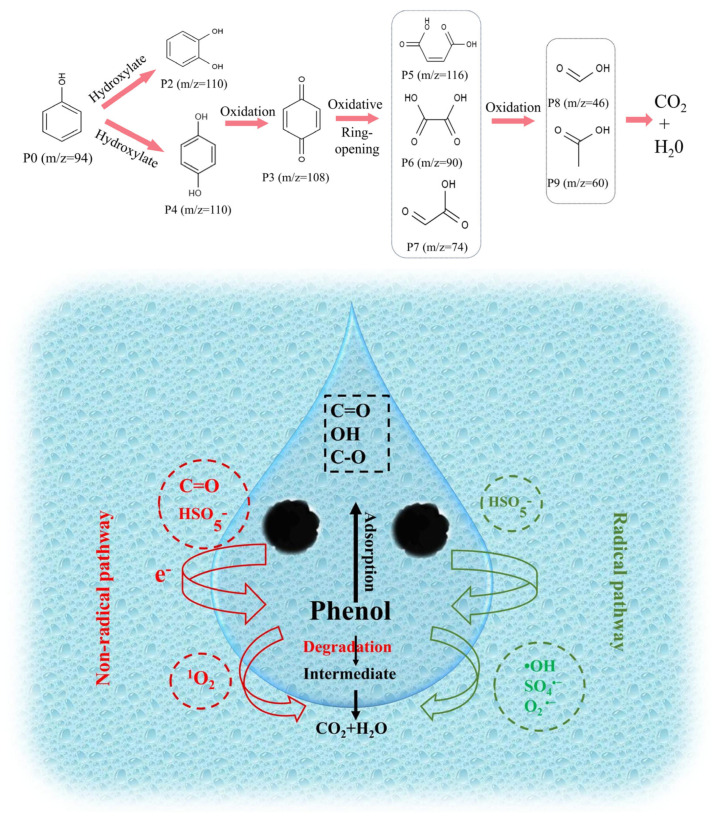
Diagram of the phenol degradation pathway and mechanism.

To identify the intermediate products and elucidate the corresponding degradation pathways during phenol removal, gas chromatography–mass spectrometry (GC–MS) was employed to monitor the oxidation process in the 900-CN/PMS system. The proposed degradation pathway for phenol is illustrated in Figure 6. The detected intermediate products primarily include p-benzoquinone, p-hydroquinone and o-cresol. During the degradation process, the •OH, SO_4_^•−^ and O_2_^•−^ radicals attack distinct positions on the benzene ring of phenol, oxidizing it to hydroxylated intermediates such as p-hydroquinone and o-cresol. Subsequently, these hydroxylated intermediates are further oxidized to quinones, such as p-benzoquinone, mediated by ^1^O_2_. Benzoquinone has the function of capturing active substances [68]. Under the action of these active substances, these quinone compounds further undergo ring-opening to form small molecule carboxylic acid compounds. These carboxylic acids may undergo further oxidation by reactive species, potentially leading to their conversion into CO_2_ and H_2_O. However, since TOC or COD measurements were not included in this study, the extent of complete mineralization cannot be fully confirmed.

## 4. Conclusions

The nitrogen-doped porous carbon composites (denoted as 900-CN) were synthesized by mixing humic acid (HA) with g-C_3_N_4_ followed by high-temperature annealing. This process resulted in a markedly altered morphology: g-C_3_N_4_ acted as a sacrificial template for the lamellar architecture, while the thermal treatment gradually generated a highly folded and porous structure. The resulting increase in specific surface area was favorable for pollutant removal. The 900-CN/PMS system achieved complete phenol removal within 60 min, exhibiting robust performance over wide pH (2–10) and temperature (20–50 °C) ranges. Notably, high degradation efficiency was maintained even at elevated phenol concentrations. Mechanistic studies revealed that although SO_4_^•−^ and •OH radicals were present in trace amounts, the system predominantly generated O_2_^•−^ and ^1^O_2_ species, which demonstrated superior oxidative capacity relative to conventional radical pathways. Phenol removal proceeded via a combination of free radical and non-free radical pathways. During degradation, phenol was decomposed into intermediates such as quinones and carboxylic acids, which were further oxidized by reactive species and ultimately mineralized to CO_2_ and H_2_O.

## Figures and Tables

**Table 1:** Comparison of material BET parameters.

Material	BET (m^2^/g)	Pore Volume (cm^3^/g)	Mean Apertur (nm)
900-HA	15.10	0.026	6.88
g-C_3_N_4_	45.13	0.16	14.21
900-CN	507.06	0.53	4.18

## Data Availability

The original contributions presented in this study are included in the article/Supplementary Material. Further inquiries can be directed to the corresponding authors.

## Supplementary Materials

The following supporting information can be downloaded at: https://www.mdpi.com/article/10.3390/molecules31060975/s1, Figure S1. Stability cycle test. Figure S2. TEM images of 900-CN. Figure S3. Raman spectra of 900-CN. Table S1. Comparison with other materials [50,69,70,71,72,73].
