# Supplementary material for: Humic Acid-Derived Porous Carbon as Peroxymonosulfate Activator for Phenol Removal
Source: Molecules. 2026 Mar 13;31(6):975. doi: 10.3390/molecules31060975 (PMC13029321; doi:10.3390/molecules31060975)
Supplement: Supplementary file 1 [file molecules-31-00975-s001.zip › molecules-4149836-supplementary.pdf]

Reusability and regeneration of 900-CN

The reusability of the 900-CN catalyst was evaluated by consecutive degradation experiments under the optimized conditions (phenol = 20 mg/L, catalyst = 0.25 g/L, PMS = 0.25 g/L, 25 °C, unadjusted pH). After each cycle, the catalyst was collected by centrifugation, washed thoroughly with ultrapure water and ethanol to remove adsorbed species, and then dried at 60 °C before the next run. As shown in Figure S1, the phenol removal efficiency remained at 100% in the first cycle, indicating the excellent initial activity of the fresh catalyst. However, in the second and third cycles, the removal efficiency declined to 62.78% and 46.38%, respectively. This gradual deactivation is likely attributed to the accumulation of reaction intermediates on the catalyst surface, which may block active sites and hinder the interaction between PMS and the catalyst, as well as possible changes in surface functional groups during the reaction.

To regenerate the spent catalyst, the used sample after the third cycle was subjected to thermal treatment at 900 °C for 1 h under N_2 atmosphere (identical to the synthesis conditions). Remarkably, the regenerated catalyst recovered 98.15% of its initial activity, achieving almost complete phenol removal within 60 min. This result demonstrates that the deactivation of 900-CN is largely reversible, and the catalyst can be effectively regenerated by simple thermal annealing, which removes adsorbed organic residues and restores the surface-active sites (e.g., nitrogen dopants and oxygen-containing groups). The excellent regenerability highlights the potential of 900-CN for practical wastewater treatment applications, where periodic regeneration can be implemented to maintain long-term catalytic performance.

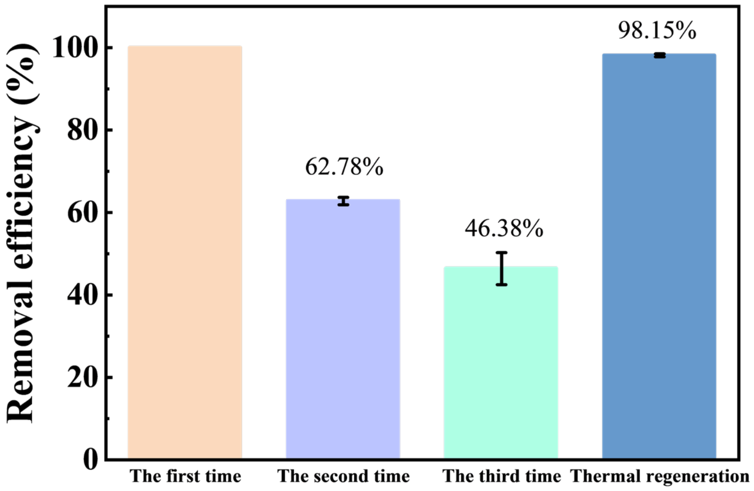

Figure S1. Stability cycle test

TEM analysis of 900-CN

To further elucidate the microstructure of the 900-CN composite at the nanoscale, transmission electron microscopy (TEM) was performed, and the results are shown in Figure S2. The low-magnification TEM image of 900-CN (Figure S2a, 50 nm) reveals a relatively rough surface morphology, with numerous fine particles observed at the edges and within the pores. These features suggest that the high specific surface area of 900-CN may enhance the contact between the catalyst and reactants, thereby improving the catalytic performance. The high-resolution TEM image (Figure S2b, 10 nm) displays blurred and discontinuous lattice fringes, indicating the absence of long-range graphitic order and confirming the amorphous nature of the carbon framework. The lack of well-defined graphite layers can be attributed to

the in situ nitrogen doping resulting from the thermal decomposition of g-C_3N_4, which introduces structural defects and disrupts the graphitic stacking. Together, the TEM and SEM observations demonstrate that the 900-CN material possesses a defect-rich, nitrogen-doped porous architecture, which is favorable for exposing abundant active sites and facilitating mass transfer during PMS activation.

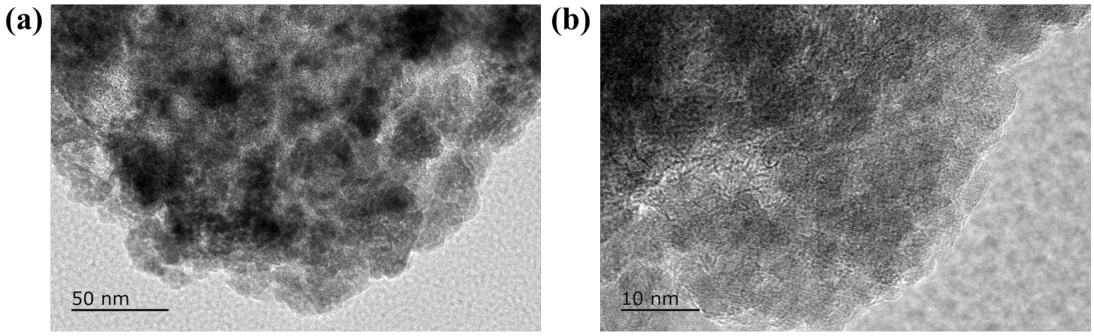

Figure S2. TEM images of 900-CN

Total organic carbon (TOC) analysis

To evaluate the mineralization degree of phenol during the catalytic oxidation, total organic carbon (TOC) was measured using a Shimadzu TOC-L CPH analyzer (Japan) equipped with an ASI-L autosampler. Samples were collected at 0 min (before PMS addition) and 90 min after reaction initiation. Immediately after sampling, each aliquot was filtered through a 0.22 μm polyethersulfone (PES) syringe filter to remove catalyst particles, and the filtrate was quenched with 0.1 mol/L sodium thiosulfate to eliminate residual PMS. The filtrate was acidified with 2 mol/L HCl to pH < 2 to eliminate inorganic carbon. The measurements were performed in the NPOC (non-purgeable organic carbon) mode, the TOC removal efficiency (mineralization rate) was calculated using the equation:

$$\mathrm{TOC\ removal(\%)} = \frac{\mathrm{TOC_0} - \mathrm{TOC_{90}}}{\mathrm{TOC_0}} \times 100$$

where TOC_0 and TOC_{90} are the TOC concentrations at 0 min and 90 min, respectively. The measured TOC removal efficiency after 90 min of reaction was 66.37%, indicating partial mineralization of phenol under the optimized conditions.

Raman spectra of 900-CN

Raman spectroscopy was employed to investigate the structural defects and graphitization degree of the optimized 900-CN catalyst. As shown in Figure S3, the Raman spectrum of 900-CN exhibits two prominent peaks at approximately 1336 cm^{-1} and 1578 cm^{-1}, corresponding to the D band (disordered carbon/defects) and G band (graphitic carbon), respectively. The intensity ratio of the D band to the G band (I_D/I_G) was calculated to be 1.2304, indicating a high density of structural defects within the carbon framework.

The high I_D/I_G value can be attributed to two synergistic factors: (i) the in situ nitrogen doping derived from the thermal decomposition of g-C_3N_4, which introduces heteroatoms into the carbon lattice and disrupts the perfect sp^2 configuration, and (ii) the sacrificial templating effect of g-C_3N_4, which generates abundant pores and edge sites during annealing. The Raman result is consistent with other characterization data: XPS confirmed multiple N configurations, and BET revealed a high specific surface area, both of which contribute to the defective nature observed in Raman. Therefore, the combination of a high I_D/I_G ratio, large surface area, and nitrogen doping collectively supports the excellent catalytic activity of 900-CN toward phenol

degradation via PMS activation.

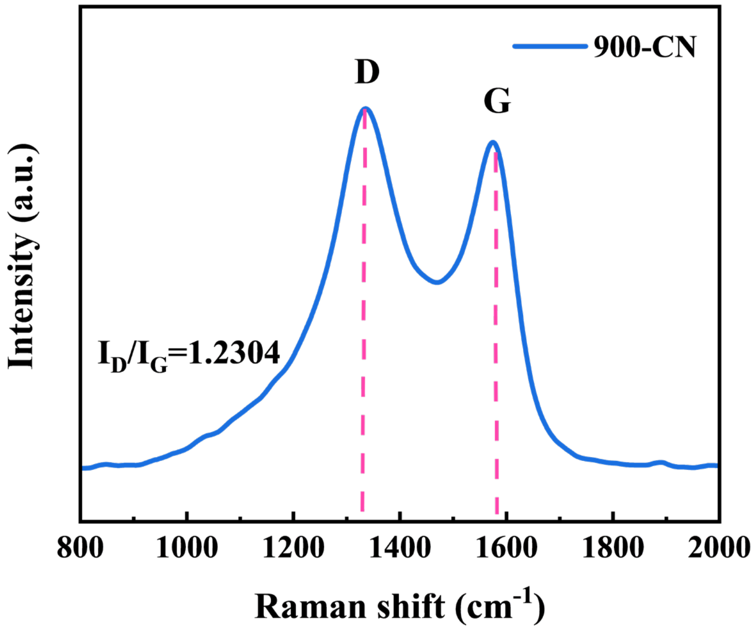

Figure S3. Raman spectra of 900-CN

Kinetic Study

The degradation kinetics of phenol by 900-CN were evaluated under optimized conditions (phenol = 20 mg/L, catalyst = 0.25 g/L, PMS = 0.25 g/L, 25 °C). Samples were collected at different time intervals and analyzed by HPLC. The pseudo-first-order kinetic model was applied using the equation:

$$\ln\left(\frac{C_0}{C_t}\right) = k \cdot t$$

Where C_0 and C_t are the phenol concentrations at time 0 and t, respectively, and k is the apparent rate constant. The degradation kinetics were fitted using the pseudo-first-order model, and a good linear fit was obtained for the initial 30 min of reaction (R^2=0.9988), yielding a rate constant k=0.096 min^{-1}. Phenol was completely removed within 45 min under the optimized conditions.

A comparison with recently reported carbon-based catalysts is presented in Table S1. The results demonstrate that 900-CN achieves complete phenol removal within 45 min under mild conditions, with a rate constant comparable to or higher than many metal-free carbon catalysts reported in the literature.

Table S1. Comparison with other materials

Catalyst	Precursor	BET (m^2/g)	Reaction conditions	Removal efficiency	k (min^{-1})	Ref.
900-CN	Humic acid+ g-C_3N_4	507	0.25 g/L cat., 0.25 g/L PMS, 25 °C	100% (45 min)	0.096	This study
NZC-S0.5	Polyaniline + SiO_2	1641	0.05 g/L cat., 1.0 g/L PMS,	99.2%	1.30	[1]

	template		25 °C	(5 min)		
PAM-0.5-700	Polyacrylamide + $NaNO_3$ template	4589	0.05 g/L cat., 1.0 g/L PMS, 25 °C	100% (1 min)	0.622	[2]
N-C-900	Tannic acid + g-C_3N_4 nanosheets	301	0.05 g/L cat., 0.65 mM PMS, 25 °C	97.6% (30 min)	0.121	[3]
CPANI-9	Polyaniline	1166	0.025 g/L cat., 0.5 mM PMS, pH 7	100% (10 min)	0.373	[4]
$FeCo_2O_4$/CNT	CNT-supported bimetallic oxide	99.8	0.3 g/L cat., 0.3 g/L PMS, 25 °C	100% (15 min)	0.30	[5]
NC-800	Humic acid + urea	_	0.2 g/L cat., 0.2 g/L PMS, 25 °C	100% (60 min)	0.067	[6]

[1] W. Zhang *et al.*, "Synergy of nitrogen doping and structural defects on hierarchically porous carbons toward catalytic oxidation via a non-radical pathway," *Carbon,* vol. 155, pp. 268-278, 2019, doi: 10.1016/j.carbon.2019.08.071.

[2] Z. Yang *et al.*, "Facile Synthesis of High-Performance Nitrogen-Doped Hierarchically Porous Carbon for Catalytic Oxidation," *ACS Sustain. Chem. Eng.,* vol. 8, no. 10, pp. 4236-4243, 2020, doi: 10.1021/acssuschemeng.9b07469.

[3] J. Qian *et al.*, "Self-assembly of tannic acid and g-C3N4 into nitrogen-doped hierarchical porous carbon for enhanced PMS activation," *Journal of Water Process Engineering,* vol. 69, 2025, doi: 10.1016/j.jwpe.2024.106616.

[4] S. Liu *et al.*, "Carbonized polyaniline activated peroxymonosulfate (PMS) for phenol degradation: Role of PMS adsorption and singlet oxygen generation," *Applied Catalysis B: Environmental,* vol. 286, 2021, doi: 10.1016/j.apcatb.2021.119921.

[5] Y. Jingjing *et al.*, "Nitrogen-doped biochar as peroxymonosulfate activator to degrade 2,4-dichlorophenol: Preparation, properties and structure–activity relationship," *Journal of Hazardous Materials,* vol. 424, 2022, doi: 10.1016/j.jhazmat.2021.127743.

[6] J. Wang *et al.*, "Carbon Nanotube-Supported FeCo2O4 as a Catalyst for an Enhanced PMS Activation of Phenol Removal," *Water,* vol. 15, no. 15, 2023, doi: 10.3390/w15152856.
